# Supplementary material for: A fault-intrusion-tolerant system and deadline-aware algorithm for scheduling scientific workflow in the cloud
Source: PeerJ Comput Sci. 2021 Nov 2;7:e747. doi: 10.7717/peerj-cs.747 (PMC8576554; doi:10.7717/peerj-cs.747)
Supplement: Supplemental Information 6 [file peerj-cs-07-747-s006.zip › Myproject1.1/docs/org/cloudbus/cloudsim/Cloudlet.html]

Cloudlet


JavaScript is disabled on your browser.


- Overview
- Package
- Class
- Use
- Tree
- Deprecated
- Index
- Help

- Prev Class
- Next Class

- Frames
- No Frames

- All Classes

- Summary:
- Nested |
- Field |
- Constr |
- Method

- Detail:
- Field |
- Constr |
- Method


org.cloudbus.cloudsim

## Class Cloudlet

- java.lang.Object
- - org.cloudbus.cloudsim.Cloudlet

- Direct Known Subclasses:
  :   NetworkCloudlet, Task

  ---

    

  ```
  public class Cloudlet
  extends java.lang.Object
  ```

  Cloudlet is an extension to the cloudlet. It stores, despite all the information encapsulated in
  the Cloudlet, the ID of the VM running it.

  Since:
  :   CloudSim Toolkit 1.0

- - ### Field Summary

    Fields

    | Modifier and Type | Field and Description |
    | --- | --- |
    | `protected double` | `accumulatedBwCost` The accumulated bw cost. |
    | `static int` | `CANCELED` The Cloudlet has been canceled. |
    | `protected double` | `costPerBw` The cost per bw. |
    | `static int` | `CREATED` The Cloudlet has been created and added to the CloudletList object. |
    | `static int` | `FAILED` The Cloudlet is failed. |
    | `static int` | `FAILED_RESOURCE_UNAVAILABLE` The cloudlet has failed due to a resource failure. |
    | `static int` | `INEXEC` The Cloudlet is in execution in a Cloud node. |
    | `static int` | `PAUSED` The Cloudlet has been paused. |
    | `static int` | `QUEUED` The Cloudlet has moved to a Cloud node. |
    | `static int` | `READY` The Cloudlet has been assigned to a CloudResource object as planned. |
    | `static int` | `RESUMED` The Cloudlet has been resumed from PAUSED state. |
    | `static int` | `SUCCESS` The Cloudlet has been executed successfully. |
    | `protected int` | `vmId` The vm id. |
  - ### Constructor Summary

    Constructors

    Constructor and Description

    `Cloudlet(int cloudletId,
    long cloudletLength,
    int pesNumber,
    long cloudletFileSize,
    long cloudletOutputSize,
    UtilizationModel utilizationModelCpu,
    UtilizationModel utilizationModelRam,
    UtilizationModel utilizationModelBw)`

    Allocates a new Cloudlet object.

    `Cloudlet(int cloudletId,
    long cloudletLength,
    int pesNumber,
    long cloudletFileSize,
    long cloudletOutputSize,
    UtilizationModel utilizationModelCpu,
    UtilizationModel utilizationModelRam,
    UtilizationModel utilizationModelBw,
    boolean record)`

    Allocates a new Cloudlet object.

    `Cloudlet(int cloudletId,
    long cloudletLength,
    int pesNumber,
    long cloudletFileSize,
    long cloudletOutputSize,
    UtilizationModel utilizationModelCpu,
    UtilizationModel utilizationModelRam,
    UtilizationModel utilizationModelBw,
    boolean record,
    java.util.List<java.lang.String> fileList)`

    Allocates a new Cloudlet object.

    `Cloudlet(int cloudletId,
    long cloudletLength,
    int pesNumber,
    long cloudletFileSize,
    long cloudletOutputSize,
    UtilizationModel utilizationModelCpu,
    UtilizationModel utilizationModelRam,
    UtilizationModel utilizationModelBw,
    java.util.List<java.lang.String> fileList)`

    Allocates a new Cloudlet object.
  - ### Method Summary

    Methods

    | Modifier and Type | Method and Description |
    | --- | --- |
    | `boolean` | `addRequiredFile(java.lang.String fileName)` Adds the required filename to the list. |
    | `boolean` | `deleteRequiredFile(java.lang.String filename)` Deletes the given filename from the list. |
    | `double` | `getActualCPUTime()` Returns the time the Cloudlet actually run. |
    | `double` | `getActualCPUTime(int resId)` Gets the total execution time of this Cloudlet in a given CloudResource ID. |
    | `int[]` | `getAllResourceId()` Gets all the CloudResource IDs that executed this Cloudlet. |
    | `java.lang.String[]` | `getAllResourceName()` Gets all the CloudResource names that executed this Cloudlet. |
    | `int` | `getClassType()` Gets the classtype or priority of this Cloudlet for scheduling on a resource. |
    | `long` | `getCloudletFileSize()` Gets the input file size of this Cloudlet BEFORE submitting to a CloudResource. |
    | `long` | `getCloudletFinishedSoFar()` Gets the length of this Cloudlet that has been executed so far from the latest CloudResource. |
    | `long` | `getCloudletFinishedSoFar(int resId)` Gets the length of this Cloudlet that has been executed so far in a given CloudResource ID. |
    | `java.lang.String` | `getCloudletHistory()` Gets the history of this Cloudlet. |
    | `int` | `getCloudletId()` Gets the ID of this Cloudlet. |
    | `long` | `getCloudletLength()` Gets the length of this Cloudlet. |
    | `long` | `getCloudletOutputSize()` Gets the output size of this Cloudlet AFTER submitting and executing to a CloudResource. |
    | `int` | `getCloudletStatus()` Gets the status code of this Cloudlet. |
    | `java.lang.String` | `getCloudletStatusString()` Gets the string representation of the current Cloudlet status code. |
    | `long` | `getCloudletTotalLength()` Gets the total length (across all PEs) of this Cloudlet. |
    | `double` | `getCostPerSec()` Gets the cost running this Cloudlet in the latest CloudResource. |
    | `double` | `getCostPerSec(int resId)` Gets the cost running this Cloudlet in a given CloudResource ID. |
    | `double` | `getExecStartTime()` Gets the latest execution start time. |
    | `double` | `getFinishTime()` Gets the finish time of this Cloudlet in a CloudResource. |
    | `int` | `getNetServiceLevel()` Gets the network service level for sending this cloudlet over a network. |
    | `int` | `getNumberOfPes()` Gets the number of PEs required to run this Cloudlet. |
    | `double` | `getProcessingCost()` Gets the total cost of processing or executing this Cloudlet Processing Cost = input data transfer + processing cost + output transfer cost . |
    | `java.util.List<java.lang.String>` | `getRequiredFiles()` Gets the required files. |
    | `int` | `getReservationId()` Gets the reservation ID that owns this Cloudlet. |
    | `org.cloudbus.cloudsim.Cloudlet.Resource` | `getResourceById(int resourceId)` Gets the resource by id. |
    | `int` | `getResourceId()` Gets the latest resource ID that processes this Cloudlet. |
    | `java.lang.String` | `getResourceName(int resId)` Gets the CloudResource name based on its ID. |
    | `int` | `getStatus()` Get the status of the Cloudlet. |
    | `static java.lang.String` | `getStatusString(int status)` Gets the string representation of the given Cloudlet status code. |
    | `double` | `getSubmissionTime()` Gets the submission or arrival time of this Cloudlet from the latest CloudResource. |
    | `double` | `getSubmissionTime(int resId)` Gets the submission or arrival time of this Cloudlet in the given CloudResource ID. |
    | `int` | `getUserId()` Gets the user or owner ID of this Cloudlet. |
    | `UtilizationModel` | `getUtilizationModelBw()` Gets the utilization model bw. |
    | `UtilizationModel` | `getUtilizationModelCpu()` Gets the utilization model cpu. |
    | `UtilizationModel` | `getUtilizationModelRam()` Gets the utilization model ram. |
    | `double` | `getUtilizationOfBw(double time)` Gets the utilization of bw. |
    | `double` | `getUtilizationOfCpu(double time)` Gets the total utilization of cpu. |
    | `double` | `getUtilizationOfRam(double time)` Gets the utilization of memory. |
    | `int` | `getVmId()` Gets the ID of the VM that will run this Cloudlet. |
    | `double` | `getWaitingTime()` Gets the waiting time of this cloudlet executed on a resource. |
    | `double` | `getWallClockTime()` Gets the time of this Cloudlet resides in the latest CloudResource (from arrival time until departure time). |
    | `double` | `getWallClockTime(int resId)` Gets the time of this Cloudlet resides in a given CloudResource ID (from arrival time until departure time). |
    | `boolean` | `hasReserved()` Checks whether this Cloudlet is submitted by reserving or not. |
    | `boolean` | `isFinished()` Checks whether this Cloudlet has finished execution or not. |
    | `boolean` | `requiresFiles()` Checks whether this cloudlet requires any files or not. |
    | `boolean` | `setClassType(int classType)` Sets the classType or priority of this Cloudlet for scheduling on a resource. |
    | `void` | `setCloudletFinishedSoFar(long length)` Sets the length of this Cloudlet that has been executed so far. |
    | `boolean` | `setCloudletLength(long cloudletLength)` Sets the length or size (in MI) of this Cloudlet to be executed in a CloudResource. |
    | `void` | `setCloudletStatus(int newStatus)` Sets the status code of this Cloudlet. |
    | `void` | `setExecParam(double wallTime, double actualTime)` Sets this Cloudlet's execution parameters. |
    | `void` | `setExecStartTime(double clockTime)` Sets the execution start time of this Cloudlet inside a CloudResource. |
    | `boolean` | `setNetServiceLevel(int netServiceLevel)` Sets the network service level for sending this cloudlet over a network. |
    | `boolean` | `setNumberOfPes(int numberOfPes)` Sets the number of PEs required to run this Cloudlet. |
    | `protected void` | `setRequiredFiles(java.util.List<java.lang.String> requiredFiles)` Sets the required files. |
    | `boolean` | `setReservationId(int resId)` Sets the id of the reservation made for this cloudlet. |
    | `void` | `setResourceParameter(int resourceID, double cost)` Sets the resource parameters for which this Cloudlet is going to be executed. |
    | `void` | `setResourceParameter(int resourceID, double costPerCPU, double costPerBw)` Sets the resource parameters for which this Cloudlet is going to be executed. |
    | `void` | `setSubmissionTime(double clockTime)` Sets the submission or arrival time of this Cloudlet into a CloudResource. |
    | `void` | `setUserId(int id)` Sets the user or owner ID of this Cloudlet. |
    | `void` | `setUtilizationModelBw(UtilizationModel utilizationModelBw)` Sets the utilization model bw. |
    | `void` | `setUtilizationModelCpu(UtilizationModel utilizationModelCpu)` Sets the utilization model cpu. |
    | `void` | `setUtilizationModelRam(UtilizationModel utilizationModelRam)` Sets the utilization model ram. |
    | `void` | `setVmId(int vmId)` Sets the ID of the VM that will run this Cloudlet. |
    | `protected void` | `write(java.lang.String str)` Writes this particular history transaction of this Cloudlet into a log. |

    - ### Methods inherited from class java.lang.Object

      `clone, equals, finalize, getClass, hashCode, notify, notifyAll, toString, wait, wait, wait`

- - ### Field Detail


    - #### CREATED

      ```
      public static final int CREATED
      ```

      The Cloudlet has been created and added to the CloudletList object.

      See Also:
      :   Constant Field Values


    - #### READY

      ```
      public static final int READY
      ```

      The Cloudlet has been assigned to a CloudResource object as planned.

      See Also:
      :   Constant Field Values


    - #### QUEUED

      ```
      public static final int QUEUED
      ```

      The Cloudlet has moved to a Cloud node.

      See Also:
      :   Constant Field Values


    - #### INEXEC

      ```
      public static final int INEXEC
      ```

      The Cloudlet is in execution in a Cloud node.

      See Also:
      :   Constant Field Values


    - #### SUCCESS

      ```
      public static final int SUCCESS
      ```

      The Cloudlet has been executed successfully.

      See Also:
      :   Constant Field Values


    - #### FAILED

      ```
      public static final int FAILED
      ```

      The Cloudlet is failed.

      See Also:
      :   Constant Field Values


    - #### CANCELED

      ```
      public static final int CANCELED
      ```

      The Cloudlet has been canceled.

      See Also:
      :   Constant Field Values


    - #### PAUSED

      ```
      public static final int PAUSED
      ```

      The Cloudlet has been paused. It can be resumed by changing the status into RESUMED.

      See Also:
      :   Constant Field Values


    - #### RESUMED

      ```
      public static final int RESUMED
      ```

      The Cloudlet has been resumed from PAUSED state.

      See Also:
      :   Constant Field Values


    - #### FAILED\_RESOURCE\_UNAVAILABLE

      ```
      public static final int FAILED_RESOURCE_UNAVAILABLE
      ```

      The cloudlet has failed due to a resource failure.

      See Also:
      :   Constant Field Values


    - #### vmId

      ```
      protected int vmId
      ```

      The vm id.


    - #### costPerBw

      ```
      protected double costPerBw
      ```

      The cost per bw.


    - #### accumulatedBwCost

      ```
      protected double accumulatedBwCost
      ```

      The accumulated bw cost.
  - ### Constructor Detail


    - #### Cloudlet

      ```
      public Cloudlet(int cloudletId,
              long cloudletLength,
              int pesNumber,
              long cloudletFileSize,
              long cloudletOutputSize,
              UtilizationModel utilizationModelCpu,
              UtilizationModel utilizationModelRam,
              UtilizationModel utilizationModelBw)
      ```

      Allocates a new Cloudlet object. The Cloudlet length, input and output file sizes should be
      greater than or equal to 1. By default this constructor sets the history of this object.

      Parameters:
      :   `cloudletId` - the unique ID of this Cloudlet
      :   `cloudletLength` - the length or size (in MI) of this cloudlet to be executed in a
          PowerDatacenter
      :   `cloudletFileSize` - the file size (in byte) of this cloudlet BEFORE submitting
          to a PowerDatacenter
      :   `cloudletOutputSize` - the file size (in byte) of this cloudlet AFTER finish
          executing by a PowerDatacenter
      :   `pesNumber` - the pes number
      :   `utilizationModelCpu` - the utilization model cpu
      :   `utilizationModelRam` - the utilization model ram
      :   `utilizationModelBw` - the utilization model bw


    - #### Cloudlet

      ```
      public Cloudlet(int cloudletId,
              long cloudletLength,
              int pesNumber,
              long cloudletFileSize,
              long cloudletOutputSize,
              UtilizationModel utilizationModelCpu,
              UtilizationModel utilizationModelRam,
              UtilizationModel utilizationModelBw,
              boolean record,
              java.util.List<java.lang.String> fileList)
      ```

      Allocates a new Cloudlet object. The Cloudlet length, input and output file sizes should be
      greater than or equal to 1.

      Parameters:
      :   `cloudletId` - the unique ID of this cloudlet
      :   `cloudletLength` - the length or size (in MI) of this cloudlet to be executed in a
          PowerDatacenter
      :   `cloudletFileSize` - the file size (in byte) of this cloudlet BEFORE submitting
          to a PowerDatacenter
      :   `cloudletOutputSize` - the file size (in byte) of this cloudlet AFTER finish
          executing by a PowerDatacenter
      :   `record` - record the history of this object or not
      :   `fileList` - list of files required by this cloudlet
      :   `pesNumber` - the pes number
      :   `utilizationModelCpu` - the utilization model cpu
      :   `utilizationModelRam` - the utilization model ram
      :   `utilizationModelBw` - the utilization model bw


    - #### Cloudlet

      ```
      public Cloudlet(int cloudletId,
              long cloudletLength,
              int pesNumber,
              long cloudletFileSize,
              long cloudletOutputSize,
              UtilizationModel utilizationModelCpu,
              UtilizationModel utilizationModelRam,
              UtilizationModel utilizationModelBw,
              java.util.List<java.lang.String> fileList)
      ```

      Allocates a new Cloudlet object. The Cloudlet length, input and output file sizes should be
      greater than or equal to 1. By default this constructor sets the history of this object.

      Parameters:
      :   `cloudletId` - the unique ID of this Cloudlet
      :   `cloudletLength` - the length or size (in MI) of this cloudlet to be executed in a
          PowerDatacenter
      :   `cloudletFileSize` - the file size (in byte) of this cloudlet BEFORE submitting
          to a PowerDatacenter
      :   `cloudletOutputSize` - the file size (in byte) of this cloudlet AFTER finish
          executing by a PowerDatacenter
      :   `fileList` - list of files required by this cloudlet
      :   `pesNumber` - the pes number
      :   `utilizationModelCpu` - the utilization model cpu
      :   `utilizationModelRam` - the utilization model ram
      :   `utilizationModelBw` - the utilization model bw


    - #### Cloudlet

      ```
      public Cloudlet(int cloudletId,
              long cloudletLength,
              int pesNumber,
              long cloudletFileSize,
              long cloudletOutputSize,
              UtilizationModel utilizationModelCpu,
              UtilizationModel utilizationModelRam,
              UtilizationModel utilizationModelBw,
              boolean record)
      ```

      Allocates a new Cloudlet object. The Cloudlet length, input and output file sizes should be
      greater than or equal to 1.

      Parameters:
      :   `cloudletId` - the unique ID of this cloudlet
      :   `cloudletLength` - the length or size (in MI) of this cloudlet to be executed in a
          PowerDatacenter
      :   `cloudletFileSize` - the file size (in byte) of this cloudlet BEFORE submitting
          to a PowerDatacenter
      :   `cloudletOutputSize` - the file size (in byte) of this cloudlet AFTER finish
          executing by a PowerDatacenter
      :   `record` - record the history of this object or not
      :   `pesNumber` - the pes number
      :   `utilizationModelCpu` - the utilization model cpu
      :   `utilizationModelRam` - the utilization model ram
      :   `utilizationModelBw` - the utilization model bw
  - ### Method Detail


    - #### setReservationId

      ```
      public boolean setReservationId(int resId)
      ```

      Sets the id of the reservation made for this cloudlet.

      Parameters:
      :   `resId` - the reservation ID

      Returns:
      :   true if the ID has successfully been set or false otherwise.


    - #### getReservationId

      ```
      public int getReservationId()
      ```

      Gets the reservation ID that owns this Cloudlet.

      Returns:
      :   a reservation ID


    - #### hasReserved

      ```
      public boolean hasReserved()
      ```

      Checks whether this Cloudlet is submitted by reserving or not.

      Returns:
      :   true if this Cloudlet has reserved before, false otherwise


    - #### setCloudletLength

      ```
      public boolean setCloudletLength(long cloudletLength)
      ```

      Sets the length or size (in MI) of this Cloudlet to be executed in a CloudResource. This
      Cloudlet length is calculated for 1 Pe only not the total length.

      Parameters:
      :   `cloudletLength` - the length or size (in MI) of this Cloudlet to be executed in a
          CloudResource

      Returns:
      :   true if it is successful, false otherwise


    - #### setNetServiceLevel

      ```
      public boolean setNetServiceLevel(int netServiceLevel)
      ```

      Sets the network service level for sending this cloudlet over a network.

      Parameters:
      :   `netServiceLevel` - determines the kind of service this cloudlet receives in the network
          (applicable to selected PacketScheduler class only)

      Returns:
      :   `true` if successful.


    - #### getNetServiceLevel

      ```
      public int getNetServiceLevel()
      ```

      Gets the network service level for sending this cloudlet over a network.

      Returns:
      :   the network service level


    - #### getWaitingTime

      ```
      public double getWaitingTime()
      ```

      Gets the waiting time of this cloudlet executed on a resource.

      Returns:
      :   the waiting time


    - #### setClassType

      ```
      public boolean setClassType(int classType)
      ```

      Sets the classType or priority of this Cloudlet for scheduling on a resource.

      Parameters:
      :   `classType` - classType of this Cloudlet

      Returns:
      :   true if it is successful, false otherwise


    - #### getClassType

      ```
      public int getClassType()
      ```

      Gets the classtype or priority of this Cloudlet for scheduling on a resource.

      Returns:
      :   classtype of this cloudlet


    - #### setNumberOfPes

      ```
      public boolean setNumberOfPes(int numberOfPes)
      ```

      Sets the number of PEs required to run this Cloudlet.   
      NOTE: The Cloudlet length is computed only for 1 Pe for simplicity.   
      For example, this Cloudlet has a length of 500 MI and requires 2 PEs. This means each Pe will
      execute 500 MI of this Cloudlet.

      Parameters:
      :   `numberOfPes` - number of Pe

      Returns:
      :   true if it is successful, false otherwise


    - #### getNumberOfPes

      ```
      public int getNumberOfPes()
      ```

      Gets the number of PEs required to run this Cloudlet.

      Returns:
      :   number of PEs


    - #### getCloudletHistory

      ```
      public java.lang.String getCloudletHistory()
      ```

      Gets the history of this Cloudlet. The layout of this history is in a readable table column
      with time and description as headers.

      Returns:
      :   a String containing the history of this Cloudlet object.


    - #### getCloudletFinishedSoFar

      ```
      public long getCloudletFinishedSoFar()
      ```

      Gets the length of this Cloudlet that has been executed so far from the latest CloudResource.
      This method is useful when trying to move this Cloudlet into different CloudResources or to
      cancel it.

      Returns:
      :   the length of a partially executed Cloudlet or the full Cloudlet length if it is
          completed


    - #### isFinished

      ```
      public boolean isFinished()
      ```

      Checks whether this Cloudlet has finished execution or not.

      Returns:
      :   true if this Cloudlet has finished execution, false otherwise


    - #### setCloudletFinishedSoFar

      ```
      public void setCloudletFinishedSoFar(long length)
      ```

      Sets the length of this Cloudlet that has been executed so far. This method is used by
      ResCloudlet class when an application is decided to cancel or to move this Cloudlet into
      different CloudResources.

      Parameters:
      :   `length` - length of this Cloudlet

      See Also:
      :   `gridsim.AllocPolicy`,
          `gridsim.ResCloudlet`


    - #### setUserId

      ```
      public void setUserId(int id)
      ```

      Sets the user or owner ID of this Cloudlet. It is VERY important to set the user ID,
      otherwise this Cloudlet will not be executed in a CloudResource.

      Parameters:
      :   `id` - the user ID


    - #### getUserId

      ```
      public int getUserId()
      ```

      Gets the user or owner ID of this Cloudlet.

      Returns:
      :   the user ID or -1 if the user ID has not been set before


    - #### getResourceId

      ```
      public int getResourceId()
      ```

      Gets the latest resource ID that processes this Cloudlet.

      Returns:
      :   the resource ID or -1 if none


    - #### getCloudletFileSize

      ```
      public long getCloudletFileSize()
      ```

      Gets the input file size of this Cloudlet BEFORE submitting to a CloudResource.

      Returns:
      :   the input file size of this Cloudlet


    - #### getCloudletOutputSize

      ```
      public long getCloudletOutputSize()
      ```

      Gets the output size of this Cloudlet AFTER submitting and executing to a
      CloudResource.

      Returns:
      :   the Cloudlet output file size


    - #### setResourceParameter

      ```
      public void setResourceParameter(int resourceID,
                              double cost)
      ```

      Sets the resource parameters for which this Cloudlet is going to be executed.   
      NOTE: This method should be called only by a resource entity, not the user or owner
      of this Cloudlet.

      Parameters:
      :   `resourceID` - the CloudResource ID
      :   `cost` - the cost running this CloudResource per second


    - #### setSubmissionTime

      ```
      public void setSubmissionTime(double clockTime)
      ```

      Sets the submission or arrival time of this Cloudlet into a CloudResource.

      Parameters:
      :   `clockTime` - the submission time


    - #### getSubmissionTime

      ```
      public double getSubmissionTime()
      ```

      Gets the submission or arrival time of this Cloudlet from the latest CloudResource.

      Returns:
      :   the submission time or 0.0 if none


    - #### setExecStartTime

      ```
      public void setExecStartTime(double clockTime)
      ```

      Sets the execution start time of this Cloudlet inside a CloudResource. **NOTE:** With new
      functionalities, such as being able to cancel / to pause / to resume this Cloudlet, the
      execution start time only holds the latest one. Meaning, all previous execution start time
      are ignored.

      Parameters:
      :   `clockTime` - the latest execution start time


    - #### getExecStartTime

      ```
      public double getExecStartTime()
      ```

      Gets the latest execution start time.

      Returns:
      :   the latest execution start time


    - #### setExecParam

      ```
      public void setExecParam(double wallTime,
                      double actualTime)
      ```

      Sets this Cloudlet's execution parameters. These parameters are set by the CloudResource
      before departure or sending back to the original Cloudlet's owner.

      Parameters:
      :   `wallTime` - the time of this Cloudlet resides in a CloudResource (from arrival time until
          departure time).
      :   `actualTime` - the total execution time of this Cloudlet in a CloudResource.


    - #### setCloudletStatus

      ```
      public void setCloudletStatus(int newStatus)
                             throws java.lang.Exception
      ```

      Sets the status code of this Cloudlet.

      Parameters:
      :   `newStatus` - the status code of this Cloudlet

      Throws:
      :   `java.lang.Exception` - Invalid range of Cloudlet status


    - #### getCloudletStatus

      ```
      public int getCloudletStatus()
      ```

      Gets the status code of this Cloudlet.

      Returns:
      :   the status code of this Cloudlet


    - #### getCloudletStatusString

      ```
      public java.lang.String getCloudletStatusString()
      ```

      Gets the string representation of the current Cloudlet status code.

      Returns:
      :   the Cloudlet status code as a string or null if the status code is unknown


    - #### getStatusString

      ```
      public static java.lang.String getStatusString(int status)
      ```

      Gets the string representation of the given Cloudlet status code.

      Parameters:
      :   `status` - the Cloudlet status code

      Returns:
      :   the Cloudlet status code as a string or null if the status code is unknown


    - #### getCloudletLength

      ```
      public long getCloudletLength()
      ```

      Gets the length of this Cloudlet.

      Returns:
      :   the length of this Cloudlet


    - #### getCloudletTotalLength

      ```
      public long getCloudletTotalLength()
      ```

      Gets the total length (across all PEs) of this Cloudlet.

      Returns:
      :   the total length of this Cloudlet


    - #### getCostPerSec

      ```
      public double getCostPerSec()
      ```

      Gets the cost running this Cloudlet in the latest CloudResource.

      Returns:
      :   the cost associated with running this Cloudlet or 0.0 if none


    - #### getWallClockTime

      ```
      public double getWallClockTime()
      ```

      Gets the time of this Cloudlet resides in the latest CloudResource (from arrival time until
      departure time).

      Returns:
      :   the time of this Cloudlet resides in a CloudResource


    - #### getAllResourceName

      ```
      public java.lang.String[] getAllResourceName()
      ```

      Gets all the CloudResource names that executed this Cloudlet.

      Returns:
      :   an array of CloudResource names or null if it has none


    - #### getAllResourceId

      ```
      public int[] getAllResourceId()
      ```

      Gets all the CloudResource IDs that executed this Cloudlet.

      Returns:
      :   an array of CloudResource IDs or null if it has none


    - #### getActualCPUTime

      ```
      public double getActualCPUTime(int resId)
      ```

      Gets the total execution time of this Cloudlet in a given CloudResource ID.

      Parameters:
      :   `resId` - a CloudResource entity ID

      Returns:
      :   the total execution time of this Cloudlet in a CloudResource or 0.0 if not
          found


    - #### getCostPerSec

      ```
      public double getCostPerSec(int resId)
      ```

      Gets the cost running this Cloudlet in a given CloudResource ID.

      Parameters:
      :   `resId` - a CloudResource entity ID

      Returns:
      :   the cost associated with running this Cloudlet or 0.0 if not found


    - #### getCloudletFinishedSoFar

      ```
      public long getCloudletFinishedSoFar(int resId)
      ```

      Gets the length of this Cloudlet that has been executed so far in a given CloudResource ID.
      This method is useful when trying to move this Cloudlet into different CloudResources or to
      cancel it.

      Parameters:
      :   `resId` - a CloudResource entity ID

      Returns:
      :   the length of a partially executed Cloudlet or the full Cloudlet length if it is
          completed or 0.0 if not found


    - #### getSubmissionTime

      ```
      public double getSubmissionTime(int resId)
      ```

      Gets the submission or arrival time of this Cloudlet in the given CloudResource ID.

      Parameters:
      :   `resId` - a CloudResource entity ID

      Returns:
      :   the submission time or 0.0 if not found


    - #### getWallClockTime

      ```
      public double getWallClockTime(int resId)
      ```

      Gets the time of this Cloudlet resides in a given CloudResource ID (from arrival time until
      departure time).

      Parameters:
      :   `resId` - a CloudResource entity ID

      Returns:
      :   the time of this Cloudlet resides in the CloudResource or 0.0 if not found


    - #### getResourceName

      ```
      public java.lang.String getResourceName(int resId)
      ```

      Gets the CloudResource name based on its ID.

      Parameters:
      :   `resId` - a CloudResource entity ID

      Returns:
      :   the CloudResource name or null if not found


    - #### getResourceById

      ```
      public org.cloudbus.cloudsim.Cloudlet.Resource getResourceById(int resourceId)
      ```

      Gets the resource by id.

      Parameters:
      :   `resourceId` - the resource id

      Returns:
      :   the resource by id


    - #### getFinishTime

      ```
      public double getFinishTime()
      ```

      Gets the finish time of this Cloudlet in a CloudResource.

      Returns:
      :   the finish or completion time of this Cloudlet or -1 if not finished yet.


    - #### write

      ```
      protected void write(java.lang.String str)
      ```

      Writes this particular history transaction of this Cloudlet into a log.

      Parameters:
      :   `str` - a history transaction of this Cloudlet


    - #### getStatus

      ```
      public int getStatus()
      ```

      Get the status of the Cloudlet.

      Returns:
      :   status of the Cloudlet


    - #### getCloudletId

      ```
      public int getCloudletId()
      ```

      Gets the ID of this Cloudlet.

      Returns:
      :   Cloudlet Id


    - #### getVmId

      ```
      public int getVmId()
      ```

      Gets the ID of the VM that will run this Cloudlet.

      Returns:
      :   VM Id, -1 if the Cloudlet was not assigned to a VM


    - #### setVmId

      ```
      public void setVmId(int vmId)
      ```

      Sets the ID of the VM that will run this Cloudlet.

      Parameters:
      :   `vmId` - the vm id


    - #### getActualCPUTime

      ```
      public double getActualCPUTime()
      ```

      Returns the time the Cloudlet actually run.

      Returns:
      :   time in which the Cloudlet was running


    - #### setResourceParameter

      ```
      public void setResourceParameter(int resourceID,
                              double costPerCPU,
                              double costPerBw)
      ```

      Sets the resource parameters for which this Cloudlet is going to be executed.   
      NOTE: This method should be called only by a resource entity, not the user or owner
      of this Cloudlet.

      Parameters:
      :   `resourceID` - the CloudResource ID
      :   `costPerCPU` - the cost running this Cloudlet per second
      :   `costPerBw` - the cost of data transfer to this PowerDatacenter


    - #### getProcessingCost

      ```
      public double getProcessingCost()
      ```

      Gets the total cost of processing or executing this Cloudlet
      Processing Cost = input data transfer + processing cost + output transfer cost .

      Returns:
      :   the total cost of processing Cloudlet


    - #### getRequiredFiles

      ```
      public java.util.List<java.lang.String> getRequiredFiles()
      ```

      Gets the required files.

      Returns:
      :   the required files


    - #### setRequiredFiles

      ```
      protected void setRequiredFiles(java.util.List<java.lang.String> requiredFiles)
      ```

      Sets the required files.

      Parameters:
      :   `requiredFiles` - the new required files


    - #### addRequiredFile

      ```
      public boolean addRequiredFile(java.lang.String fileName)
      ```

      Adds the required filename to the list.

      Parameters:
      :   `fileName` - the required filename

      Returns:
      :   true if succesful, false otherwise


    - #### deleteRequiredFile

      ```
      public boolean deleteRequiredFile(java.lang.String filename)
      ```

      Deletes the given filename from the list.

      Parameters:
      :   `filename` - the given filename to be deleted

      Returns:
      :   true if succesful, false otherwise


    - #### requiresFiles

      ```
      public boolean requiresFiles()
      ```

      Checks whether this cloudlet requires any files or not.

      Returns:
      :   true if required, false otherwise


    - #### getUtilizationModelCpu

      ```
      public UtilizationModel getUtilizationModelCpu()
      ```

      Gets the utilization model cpu.

      Returns:
      :   the utilization model cpu


    - #### setUtilizationModelCpu

      ```
      public void setUtilizationModelCpu(UtilizationModel utilizationModelCpu)
      ```

      Sets the utilization model cpu.

      Parameters:
      :   `utilizationModelCpu` - the new utilization model cpu


    - #### getUtilizationModelRam

      ```
      public UtilizationModel getUtilizationModelRam()
      ```

      Gets the utilization model ram.

      Returns:
      :   the utilization model ram


    - #### setUtilizationModelRam

      ```
      public void setUtilizationModelRam(UtilizationModel utilizationModelRam)
      ```

      Sets the utilization model ram.

      Parameters:
      :   `utilizationModelRam` - the new utilization model ram


    - #### getUtilizationModelBw

      ```
      public UtilizationModel getUtilizationModelBw()
      ```

      Gets the utilization model bw.

      Returns:
      :   the utilization model bw


    - #### setUtilizationModelBw

      ```
      public void setUtilizationModelBw(UtilizationModel utilizationModelBw)
      ```

      Sets the utilization model bw.

      Parameters:
      :   `utilizationModelBw` - the new utilization model bw


    - #### getUtilizationOfCpu

      ```
      public double getUtilizationOfCpu(double time)
      ```

      Gets the total utilization of cpu.

      Parameters:
      :   `time` - the time

      Returns:
      :   the utilization of cpu


    - #### getUtilizationOfRam

      ```
      public double getUtilizationOfRam(double time)
      ```

      Gets the utilization of memory.

      Parameters:
      :   `time` - the time

      Returns:
      :   the utilization of memory


    - #### getUtilizationOfBw

      ```
      public double getUtilizationOfBw(double time)
      ```

      Gets the utilization of bw.

      Parameters:
      :   `time` - the time

      Returns:
      :   the utilization of bw


- Overview
- Package
- Class
- Use
- Tree
- Deprecated
- Index
- Help

- Prev Class
- Next Class

- Frames
- No Frames

- All Classes

- Summary:
- Nested |
- Field |
- Constr |
- Method

- Detail:
- Field |
- Constr |
- Method
